# The First Complete Mitochondrial Genome of *Lecanora argopholis* and Its Phylogenetic Implications for Lichen-Forming Fungi

**DOI:** 10.3390/jof12100714

**Published:** 2026-09-23

**Authors:** Guldiyar Adil, Erfan Akberjan, Reyim Mamut

**Affiliations:** 1 College of Life Sciences and Technology, Xinjiang University, Urumqi 830017, China; 107552301017@stu.xju.edu.cn; 2 Xinjiang Administration Center of West Tianshan National Nature Reserve, Yining 835000, China; ylf998223@163.com; 3 Xinjiang West Tianshan Forest Ecosystem Observation and Research Station, Gongliu County 835400, China

**Keywords:** *Lecanora argopholis*, mitogenome, comparative analysis, genome synteny, phylogenetic analysis

## Abstract

We report the first complete mitochondrial genome of the crustose lichen-forming fungus *Lecanora argopholis*. The circular mitogenome is 48,790 bp in length, has an overall AT content of 70.4%, and contains 14 protein-coding genes (PCGs), two rRNA genes, and 29 tRNA genes. Comparative analysis of four *Lecanora* species showed that mitochondrial tRNA features were generally conserved, although limited variation in tRNA length and predicted base-pairing patterns was observed among the sampled taxa. Relative synonymous codon usage analysis of 13 shared mitochondrial PCGs revealed a similar preference for A/U-ending codons across the four species, indicating that this pattern is not unique to *L. argopholis*. Expanded synteny analysis of eight lichen-forming fungal mitogenomes identified conserved homologous blocks together with variation in mitochondrial gene order; among the sampled taxa, *L. argopholis* and *L. cinereofusca* showed the highest gene-order conservation. Phylogenetic analysis based on 14 concatenated mitochondrial PCGs from 17 lichen-forming fungi recovered *L. argopholis* as sister to *L. cinereofusca*, providing additional mitochondrial evidence relevant to the placement of *L. argopholis* within the taxonomically complex *Lecanora* s. lat. These results expand the mitogenomic resources available for *Lecanora* and provide comparative information on mitochondrial genome organization and evolution in lichen-forming fungi.

## 1. Introduction

Lichens represent highly specialized and ecologically successful symbiotic associations, primarily involving a lichen-forming fungus and one or more photosynthetic partners [1]. They colonize diverse substrates, including rocks, bark, soil, bryophytes, and plant debris, and contribute to ecosystem functioning through roles in primary succession, nutrient cycling, and environmental indication [2,3,4]. Because of their sensitivity to atmospheric and substrate conditions, lichens have also been widely used as biomonitors of environmental change and pollution [2,3].

The lichen genus *Lecanora* Ach. is one of the largest genera of lichenized fungi and shows large variation in thallus and apothecium morphology [5]. Traditionally, species delimitation and infrageneric classification in this group have relied mainly on thallus morphology, apothecial anatomy, and secondary chemistry. *Lecanora* was originally defined by traditional characteristics such as lecanorine apothecia, colorless, ellipsoid, 8-spored asci, and the presence of atranorin. In the early stages, it included species with similar morphological features, which led to the formation of one of the most species-rich and extensive groups. Subsequently, molecular biological techniques were adopted for classification, gradually clarifying the taxonomic status of species within this genus. However, recent molecular phylogenetic studies have consistently reached the same conclusion: the traditional morphological classification system of the broadly defined *Lecanora* does not align with the phylogenetic trees reconstructed from molecular sequencing data, and thus fails to objectively reflect the true evolutionary relationships among the various groups. Recently, DNA-based phylogenetic approaches have provided a better taxonomic understanding of *Lecanora* s. lat., from species to subgeneric groups and their evolutionary relationships [6,7,8,9,10,11,12]. These findings indicate that additional molecular data are still needed to better understand relationships within *Lecanora* s. lat. and to assess the phylogenetic placement of individual species in this complex lineage.

*Lecanora argopholis* (Ach.) Ach. is a crustose to subfruticose lichen with a broad Holarctic distribution [13], particularly a dominant lichen species in arid and high-altitude regions. The thallus of this species is permanent, well developed, thick, and has numerous apothecia that are always present in the thallus center. The thallus color is variable, ranging from yellowish green and pale green to green; pale brown thalli also occur in the field. It visually resembles placodioid species of *Lecanora*, but lacks usnic acid and contains atranorin. Despite its relatively wide distribution, this species remains poorly represented in genomic studies, and no complete mitogenome has yet been reported for this species.

Metagenomic and next-generation sequencing platforms are increasingly practical tools for a variety of detailed investigations of microorganisms in natural ecosystems, including lichen symbionts that are generally recalcitrant to pure culture in vitro [14,15]. Mitogenomes have become increasingly valuable in fungal evolutionary studies because they provide both relatively conserved protein-coding genes (PCGs) for phylogenetic inference and comparative genomic characters such as genome size variation, gene order, and structural rearrangements [16,17,18,19]. Mitochondrial (mt) DNA has been used extensively in evolutionary and population studies of all types of eukaryotes and is usually inherited in a uniparental manner [20]. In recent years, mitochondrial genome sequencing technology has matured, and an increasing number of complete mitochondrial genomes from lichen-forming fungi have been resolved. Researchers have conducted extensive studies on the base composition, gene structure, gene arrangement, codon usage characteristics, synteny, and phylogenetic position of lichen mitochondrial genomes, gradually clarifying the basic structural features and evolutionary patterns of lichen-forming fungal mitochondrial genomes [18,21,22,23]. These findings provide reliable molecular evidence for species classification and phylogenetic reconstruction of lichens.

In this study, we report and annotate the first complete mitochondrial genome of *L. argopholis*. We characterize its genome organization and tRNA complement, compare mitochondrial tRNA features and codon-usage patterns among four *Lecanora* species, and examine mitochondrial synteny and gene-order conservation across eight lichen-forming fungi. We further evaluate the phylogenetic position of *L. argopholis* using 14 concatenated mitochondrial PCGs from 17 taxa. These analyses expand the mitogenomic resources available for *Lecanora* and provide comparative data for assessing mitochondrial genome conservation and variation within this taxonomically complex group.

## 2. Materials and Methods

### 2.1. Sample Collection and DNA Extraction

*L. argopholis* was collected from Kabanbay Peak, Gongliu County, Yili, Xinjiang, China (83°24′47″ E, 43°01′11″ N) (Figure 1; Supplementary Table S1). Species identification was based on morphological characteristics and further supported by sequence analysis of the internal transcribed spacer (ITS) region (GenBank accession no. PV683001) [24]. The voucher specimen was deposited at the College of Life Sciences and Technology, Xinjiang University, Urumqi, China, under voucher number XT0081. Total genomic DNA was extracted from the thallus using the Ezup Column Fungi Genomic DNA Purification Kit (Sangon Biotech, Shanghai, China) according to the manufacturer’s instructions. DNA quality and concentration were assessed prior to library construction and sequencing.

### 2.2. Quality Control, Genome Assembly and Annotation

Raw reads generated on the MGI DNBSEQ platform (Shenzhen Huitong Biotechnology Co., Ltd., Shenzhen, China) were initially assessed using FastQC v0.11.9 to evaluate sequence quality, GC content, and sequence duplication levels [25]. Adapter sequences and low-quality reads were removed using fastp v0.23.2 [26]. The quality-filtered reads were then de novo assembled using GetOrganelle v1.7.4.1 [27], and the assembly graph was inspected using Bandage v0.8.1 to assess genome circularity and assembly completeness [28]. The assembled contigs were subsequently queried against the NCBI nucleotide (nt) database using BLASTn v2.15.0+ [29] to identify mitochondrial contigs and distinguish them from non-target sequences. Sequencing depth across the finalized mitogenome was calculated using SAMtools v1.19.2 [30] and visualized in IGV v2.14.1 [31] to evaluate genome-wide sequencing support (Supplementary Figure S1). The mitogenome was annotated using MITOS2 web server (http://mitos2.bioinf.uni-leipzig.de; accessed on 22 June 2026) and GeSeq (https://chlorobox.mpimp-golm.mpg.de/geseq; accessed on 22 June 2026) under mitochondrial genetic code 4 [32,33], and the resulting annotations were manually inspected and corrected where necessary using Geneious Prime 2025.2.2 (Biomatters Ltd., Auckland, New Zealand). The final circular genome map was generated using OGDRAW v1.3.1 [34].

### 2.3. tRNA Secondary Structure and Comparative Analysis

Mitochondrial tRNA genes were identified and their secondary structures were predicted using tRNAscan-SE v2.0.12 [35]. The predicted tRNA secondary structures of *L. argopholis* were visualized using VARNA v3.93 [36] and manually inspected. For comparative analysis, *L. argopholis*, *L. cinereofusca*, *L. saxigena*, and *L. strobilina* were analyzed using the same tRNAscan-SE workflow. Only confidently retained predictions were used for comparative structural analysis. Mitochondrial tRNAs were compared in terms of anticodon identity, copy number, sequence length, and predicted structural characteristics. For selected tRNAs, the arrangement of adjacent mitochondrial genes was also compared among the four species.

### 2.4. Comparative Synonymous Codon Usage Analysis

To compare mitochondrial codon usage among *L. argopholis*, *L. cinereofusca*, *L. saxigena*, and *L. strobilina*, the coding sequences of 13 shared mitochondrial PCGs (*atp6*, *atp8*, *cob*, *cox1*, *cox2*, *cox3*, *nad1*, *nad2*, *nad3*, *nad4*, *nad5*, *nad6*, and *rps3*) were extracted, with intronic regions excluded. Codon counts and relative synonymous codon usage (RSCU) values were calculated under mitochondrial genetic code 4, with stop codons excluded from the analysis. Codons with RSCU values > 1.0 were considered preferentially used. Comparative RSCU profiles were visualized using Matplotlib v3.10.6 in Python v3.10.12 [37].

### 2.5. Synteny and Gene-Order Analysis

Comparative mitochondrial synteny was analyzed among eight complete mitogenomes, including four *Lecanora* species and four related lichen-forming fungi (Supplementary Table S2). To facilitate comparisons among circular genomes, each mitogenome was normalized to a common *cox1* starting position while preserving its original orientation. Whole-mitogenome locally collinear blocks (LCBs) were identified using progressiveMauve in Mauve v2.4.0 [38]. Gene-order conservation was further assessed using 15 mitochondrial genes shared across all eight taxa, comprising 13 PCGs and the two rRNA genes (*rrnS* and *rrnL*). For each pair of taxa, conserved circular gene adjacencies were counted, and gene-order similarity was summarized using the conserved adjacency fraction and the Jaccard similarity of adjacency sets.

### 2.6. Phylogenetic Analysis

The phylogenetic position of *L. argopholis* was inferred using a concatenated dataset of 14 shared mitochondrial PCGs from 17 lichen-forming fungal taxa, including the newly assembled mitogenome of *L. argopholis* and 16 published mitogenomes retrieved from NCBI (Supplementary Table S2). Sequence extraction, concatenation, and dataset preparation were performed in PhyloSuite v1.2.3 [39]. Individual genes were aligned using MAFFT v7.505 [40], and ambiguously aligned regions were removed using Gblocks v0.91b [41]. Maximum-likelihood (ML) analysis was conducted in IQ-TREE v2.2.0 [42], with the best-fit substitution model selected by ModelFinder [43] and branch support assessed using 1000 ultrafast bootstrap replicates. *Cladonia uncialis* and *C. macilenta* were selected as outgroups because they represent Cladoniaceae outside the sampled ingroup families and have complete mitochondrial genomes available; the tree was rooted accordingly. The resulting phylogenetic tree was visualized and edited in FigTree v1.4.4 [44].

## 3. Results

### 3.1. Structural Characteristics of the Mitogenome

The complete mitogenome of *L. argopholis* was assembled as a circular DNA molecule of 48,790 bp (Figure 2). Read mapping to the finalized mitogenome showed 100% genome coverage, with an average sequencing depth of 7221× (range: 1032–8015×) (Supplementary Figure S1). A total of 45 genes were annotated, including 14 PCGs, two rRNA genes, and 29 tRNA genes. These genes were classified into seven functional categories (Table 1). The 14 PCGs comprised seven NADH dehydrogenase subunit genes (*nad1*, *nad2*, *nad3*, *nad4*, *nad4L*, *nad5*, and *nad6*), three cytochrome c oxidase genes (*cox1*, *cox2*, and *cox3*), two ATP synthase subunit genes (*atp6* and *atp8*), one cytochrome *b* gene (*cob*), and one ribosomal protein gene (*rps3*). The mitogenome comprised 34.0% A, 36.4% T, 15.1% G, and 14.5% C, corresponding to an overall AT content of 70.4%. The annotated genes were distributed on both strands of the mitogenome.

### 3.2. tRNA Genes and Secondary Structures

A total of 29 mitochondrial tRNA genes representing 22 tRNA types were identified in *L. argopholis* (Figure 3). Comparative analysis retained 107 confidently predicted tRNAs across the four *Lecanora* species, including 29 in *L. argopholis*, 26 in *L. cinereofusca*, 27 in *L. saxigena*, and 25 in *L. strobilina* (Supplementary Table S3A). Among the five focal tRNAs examined in detail, *trnS1*, *trnS2*, *trnL2*, and *trnY* showed conserved sequence lengths across the four species, whereas *trnL1* varied from 82 to 85 nt (Supplementary Table S3B). Despite the conserved lengths of several tRNAs, differences in predicted base-pairing patterns and G–U wobble pairs were observed among species. The genomic neighborhoods surrounding *trnS1*, *trnS2*, *trnL1*, and *trnL2* were conserved across the four taxa, whereas the broader genomic context surrounding *trnY* was more variable. No clear correspondence was observed between structural variation in the focal tRNAs and local differences in mitochondrial gene order.

### 3.3. Comparative Synonymous Codon Usage

Comparative RSCU analysis of 13 shared mitochondrial PCGs revealed broadly similar codon-usage profiles among the four *Lecanora* species (Figure 4; Supplementary Table S4). A total of 25, 26, 26, and 24 codons had RSCU values > 1.0 in *L. argopholis*, *L. cinereofusca*, *L. saxigena*, and *L. strobilina*, respectively. All preferentially used codons in *L. argopholis*, *L. saxigena*, and *L. strobilina* ended in A or U, whereas 25 of the 26 preferentially used codons in *L. cinereofusca* had A/U endings (Supplementary Table S4A). UUA (Leu) was preferentially used in all four species, with RSCU values ranging from 3.344 to 3.540. The corresponding values were 3.344 in *L. argopholis*, 3.353 in *L. cinereofusca*, 3.540 in *L. saxigena*, and 3.498 in *L. strobilina* (Supplementary Table S4B).

### 3.4. Mitochondrial Genome Synteny and Gene-Order Conservation

Comparative analysis of eight lichen-forming fungal mitogenomes identified 15 locally collinear blocks (LCBs) shared by at least two taxa, including nine core LCBs present in all eight mitogenomes (Figure 5; Supplementary Table S5B). These universally conserved blocks encompassed regions associated with *cox1*, *nad4*, *cob–cox2*, *nad1*, *atp6*, *nad6–atp8–rrnS*, *cox3*, *rrnL–nad2–nad3*, and *nad4L–nad5*. No reverse-oriented shared LCBs were detected among the eight mitogenomes.

Gene-order analysis based on 15 shared mitochondrial genes showed the highest pairwise conservation between *L. argopholis* and *L. cinereofusca*, which shared all 15 circular gene adjacencies (Supplementary Table S5A). *L. argopholis* shared 12 of 15 adjacencies with both *L. saxigena* and *L. strobilina*, whereas *L. saxigena* and *L. strobilina* shared 10 of 15. Four adjacencies (*cob–cox2*, *rrnS–nad6*, *rrnL–nad2*, and *nad2–nad3*) were conserved across all eight taxa, whereas *cox1–nad4*, *rps3–cob*, and *cox2–nad1* showed greater variation among the sampled mitogenomes.

### 3.5. Phylogenetic Relationships

The maximum-likelihood phylogeny based on the concatenated nucleotide sequences of 14 mitochondrial PCGs recovered *L. argopholis* within Lecanoraceae (Figure 6). *Lecanora argopholis* formed a sister relationship with *L. cinereofusca* with strong bootstrap support (BS = 97). This pair further clustered with *L. strobilina* (BS = 94), and these three taxa together with *L. saxigena* formed a well-supported clade (BS = 100). At the family level, Lecanoraceae and Parmeliaceae were recovered as sister groups with strong support (BS = 100), while *Ramalina intermedia* (Ramalinaceae) was placed outside this clade. The two *Cladonia* species formed a strongly supported clade (BS = 100) and were used to root the tree. Within Parmeliaceae, the sampled genera generally formed well-supported subclades, although support for some internal relationships was lower, including the node involving *Xanthoparmelia pulvinaris* (BS = 64) and the *Usnea subgracilis–U. ceratina* grouping (BS = 80).

## 4. Discussion

The complete mitogenome of *L. argopholis* reported here adds a new mitochondrial genome to the limited genomic resources currently available for *Lecanora*. Its gene complement, comprising 14 PCGs, two rRNA genes, and 29 tRNA genes, is broadly consistent with the conserved mitochondrial coding set reported from other lichen-forming fungi [16,17,18,19]. The comparative tRNA analysis further showed that several structural features were conserved among the four sampled taxa. In particular, *trnS1*, *trnS2*, *trnL2*, and *trnY* retained the same lengths across the four species, whereas *trnL1* varied from 82 to 85 nt. At the same time, differences in predicted base pairing and G–U wobble pairs occurred even when overall tRNA length was unchanged. These observations suggest that mitochondrial tRNA variation in the sampled taxa is relatively localized rather than accompanied by extensive structural divergence. The genomic neighborhoods of *trnS1*, *trnS2*, *trnL1*, and *trnL2* were also conserved, while a broader variation was observed around *trnY*. However, no clear correspondence was found between variation in the focal tRNA structures and local differences in mitochondrial gene order. The present data therefore do not support a simple relationship between local genome rearrangement and the structural variation in these tRNAs.

Codon-usage patterns were also broadly similar among the four sampled *Lecanora* mitogenomes. Preferentially used codons were overwhelmingly A- or U-ending, and this tendency was shared across all four taxa rather than being specific to *L. argopholis*. This pattern is consistent with the high AT content of these mitochondrial genomes and with the general influence of nucleotide composition on synonymous codon usage. UUA (Leu) was preferentially used in all four species, with RSCU values ranging from 3.344 to 3.540. Importantly, the value in *L. argopholis* (3.344) was slightly lower than those observed in the other three taxa, indicating that the elevated use of UUA is not an unusual feature of *L. argopholis*. Thus, the comparative analysis supports a shared codon-usage pattern among the sampled species rather than a species-specific bias. Because expression-level and translational data were unavailable, possible effects on translation efficiency or adaptive significance were not evaluated. Potential mitochondrial RNA editing was also not assessed because transcriptomic data were unavailable. Accordingly, the codon-usage patterns reported here reflect genomic coding sequences rather than edited mitochondrial transcripts [45].

Comparative analysis of eight lichen-forming fungal mitogenomes showed that conserved mitochondrial regions coexist with appreciable variation in gene order. Fifteen shared LCBs were identified, including nine core blocks present in all eight taxa, and no reverse-oriented shared LCBs were detected. Several conserved regions contained functionally important mitochondrial genes, including *cox1*, *nad4*, *cob–cox2*, *nad1*, *atp6*, *cox3*, and the *rrnL–nad2–nad3* region. These conserved blocks indicate that substantial portions of mitochondrial genome organization have been retained across the sampled lineages. At the same time, comparisons of circular gene adjacencies revealed clear differences among taxa. *L. argopholis* and *L. cinereofusca* shared all 15 examined adjacencies, whereas *L. argopholis* shared 12 of 15 with both *L. saxigena* and *L. strobilina*. Some junctions, including *cob–cox2*, *rrnS–nad6*, *rrnL–nad2*, and *nad2–nad3*, were conserved across all eight mitogenomes, while others were more variable. Previous studies have similarly shown that mitochondrial genomes of lichen-forming fungi can retain a broadly conserved core gene set while differing in genome organization [16,17,18,19,21,22,23]. Our results extend this pattern to *L. argopholis* and show that gene-order conservation can differ substantially even among the sampled members of Lecanoraceae. Because no reverse-oriented shared LCBs were detected, the variation observed here is better described as differences in LCB order and gene adjacency rather than as evidence for large-scale mitochondrial inversions.

The mitochondrial phylogeny provides an additional line of evidence for the placement of *L. argopholis*. The species was recovered as sister to *L. cinereofusca* with strong bootstrap support (BS = 97), and this relationship is also consistent with the complete conservation of the 15 examined circular gene adjacencies between the two mitogenomes. This correspondence is noteworthy, although gene-order similarity should not be interpreted as independent proof of phylogenetic relatedness. The four sampled Lecanoraceae taxa formed a strongly supported clade, and Lecanoraceae and Parmeliaceae were recovered as sister groups in the present mitochondrial dataset. These deeper relationships should be interpreted cautiously because the analysis was designed primarily to evaluate the position of *L. argopholis* and included limited representatives of several families. The two *Cladonia* species were used as outgroups to root the tree, and alternative outgroup configurations were not examined. More broadly, the circumscription of *Lecanora* s. lat. remains under active reassessment based on multilocus datasets [6,11]. Mitochondrial loci are inherited as a linked genomic unit and therefore represent only one component of species evolutionary history; mitochondrial and nuclear phylogenies may differ because of incomplete lineage sorting, introgression, differences in inheritance, or evolutionary rate [20]. For this reason, the sister relationship recovered here is best regarded as mitochondrial evidence that complements, rather than replaces, nuclear and multilocus systematic studies. Denser taxon sampling and combined mitochondrial and nuclear datasets will be needed to test the stability of the relationships observed here and to clarify the broader systematic position of *L. argopholis* within the *Lecanora* complex.

## 5. Conclusions

In this study, we report the first complete mitogenome of *L. argopholis*, a circular molecule of 48,790 bp containing 14 protein-coding genes, two rRNA genes, and 29 tRNA genes. Comparative analyses among the four sampled taxa revealed broadly conserved mitochondrial tRNA features with limited interspecific structural variation and a shared preference for A/U-ending synonymous codons. Expanded synteny analysis across eight lichen-forming fungal mitogenomes identified conserved homologous blocks together with variation in mitochondrial gene order, with *L. argopholis* and *L. cinereofusca* showing the highest gene-order conservation among the sampled taxa. Consistent with this pattern, mitochondrial phylogenetic analysis recovered *L. argopholis* as sister to *L. cinereofusca*. These results expand the available mitogenomic resources for *Lecanora* and provide comparative evidence for both conserved and variable features of mitochondrial genome organization in lichen-forming fungi.

## Figures and Tables

**Figure 1:**
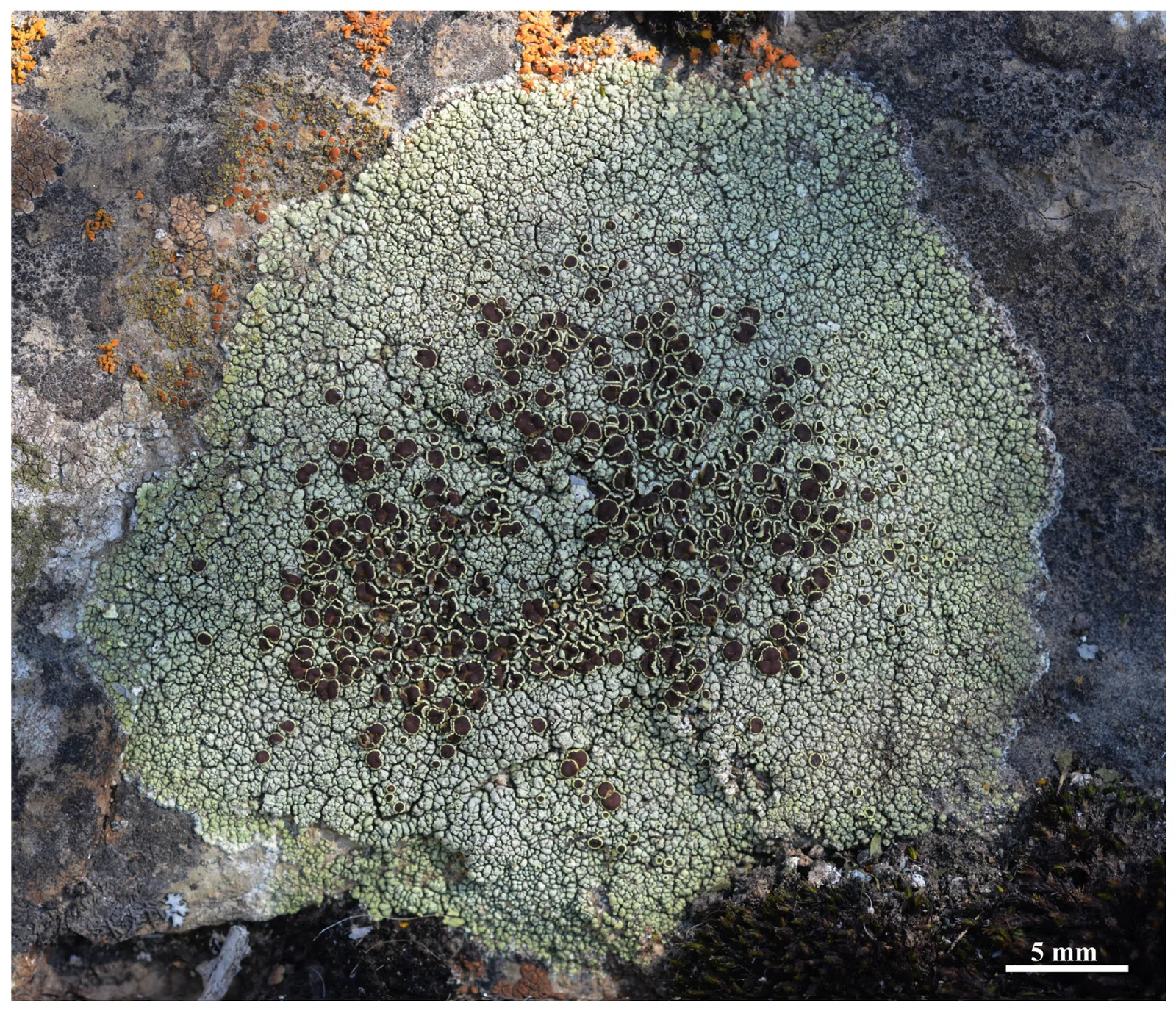
Thallus of *Lecanora argopholis* in its natural habitat. Photo by Reyim Mamut.

**Figure 2:**
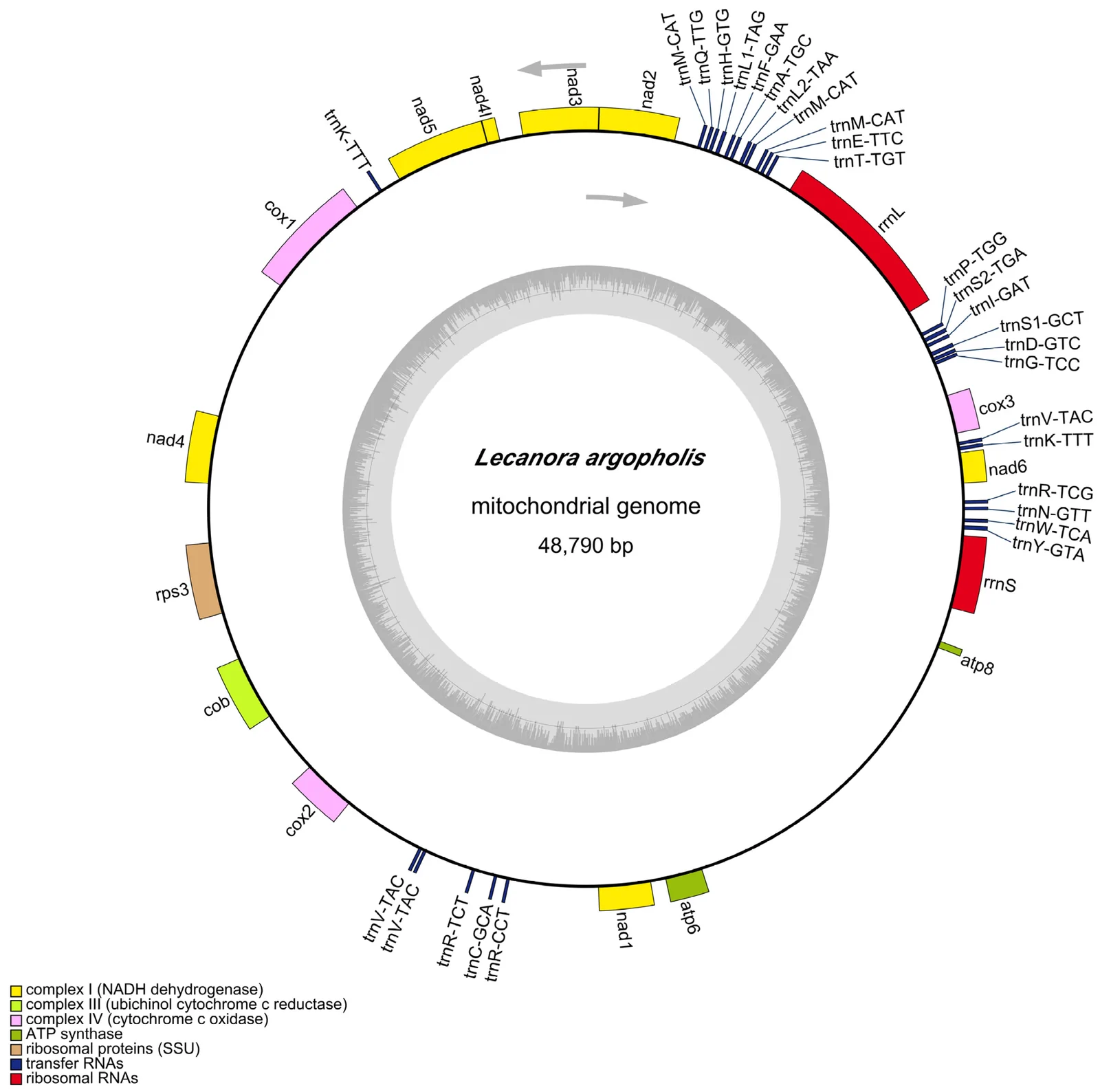
Circular map of the mitochondrial genome of *Lecanora argopholis*. Genes are color-coded according to their functional categories, as indicated in the legend. Gray arrows indicate the direction of gene transcription on the two mitochondrial strands.

**Figure 3:**
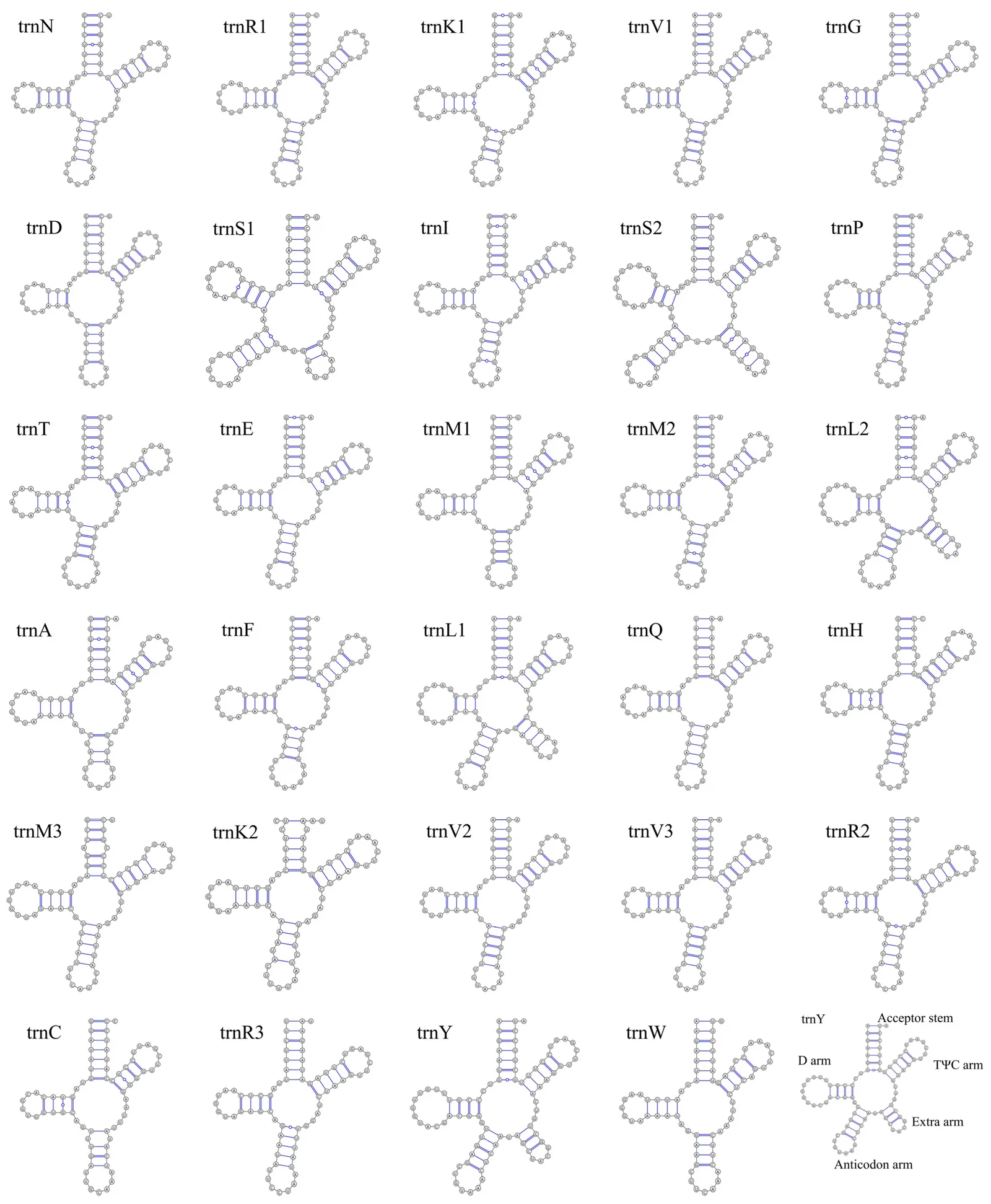
Predicted secondary structures of mitochondrial tRNAs in *Lecanora argopholis*. Twenty-nine tRNAs representing 22 tRNA types are shown. The major structural domains, including the acceptor stem, D arm, anticodon arm, TΨC arm, and extra arm, are indicated. A, U, G, and C denote adenine, uracil, guanine, and cytosine, respectively.

**Figure 4:**
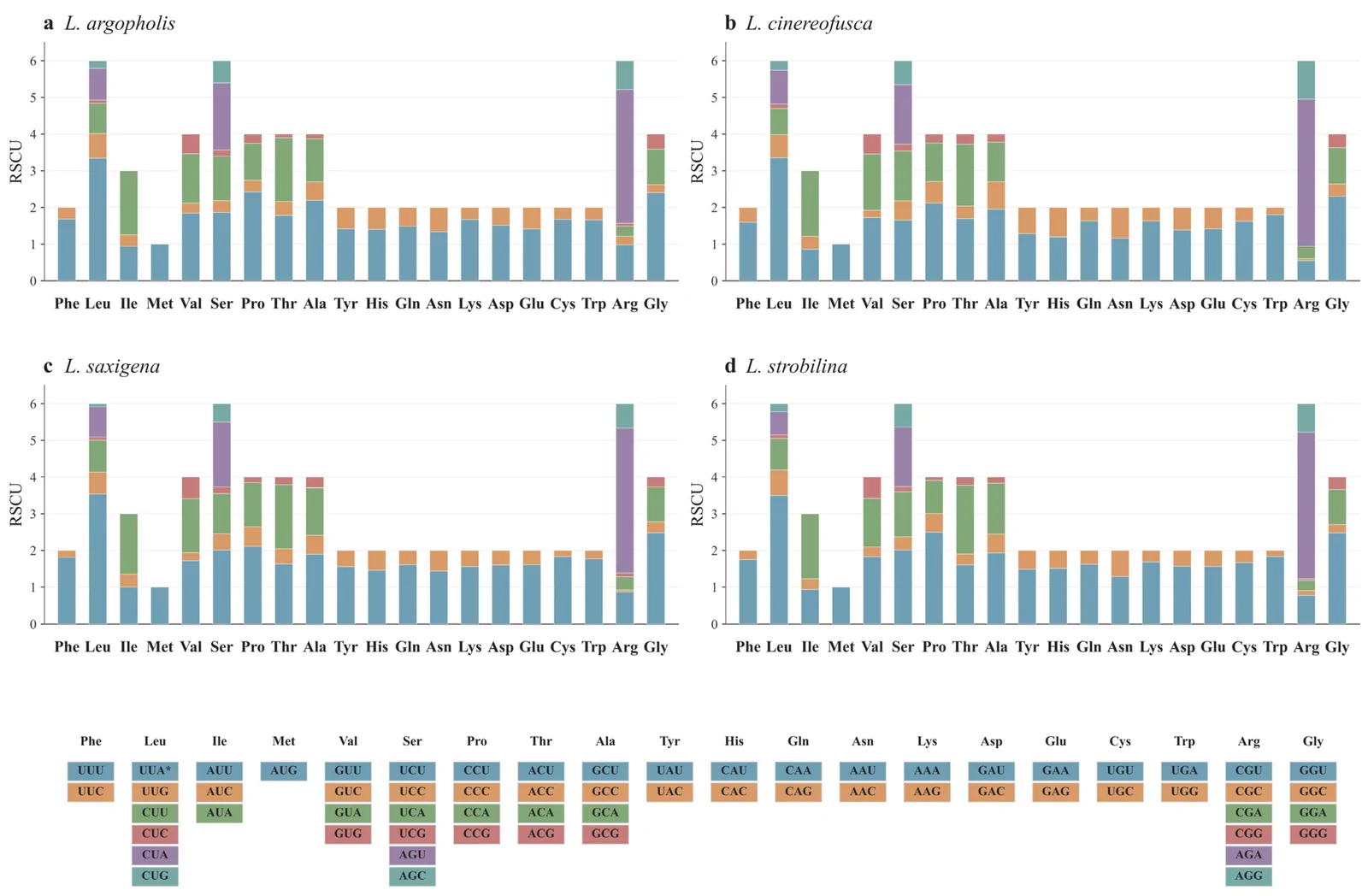
Comparative relative synonymous codon usage (RSCU) profiles of 13 shared mitochondrial protein-coding genes in four *Lecanora* species. Panels (**a**–**d**) represent *L. argopholis*, *L. cinereofusca*, *L. saxigena*, and *L. strobilina*, respectively. Stacked bars show RSCU values for synonymous codons, with codon identities indicated in the shared key below the panels. The asterisk marks UUA (Leu), which is highlighted because it is discussed in the main text and was preferentially used (RSCU > 1.0) in all four species.

**Figure 5:**
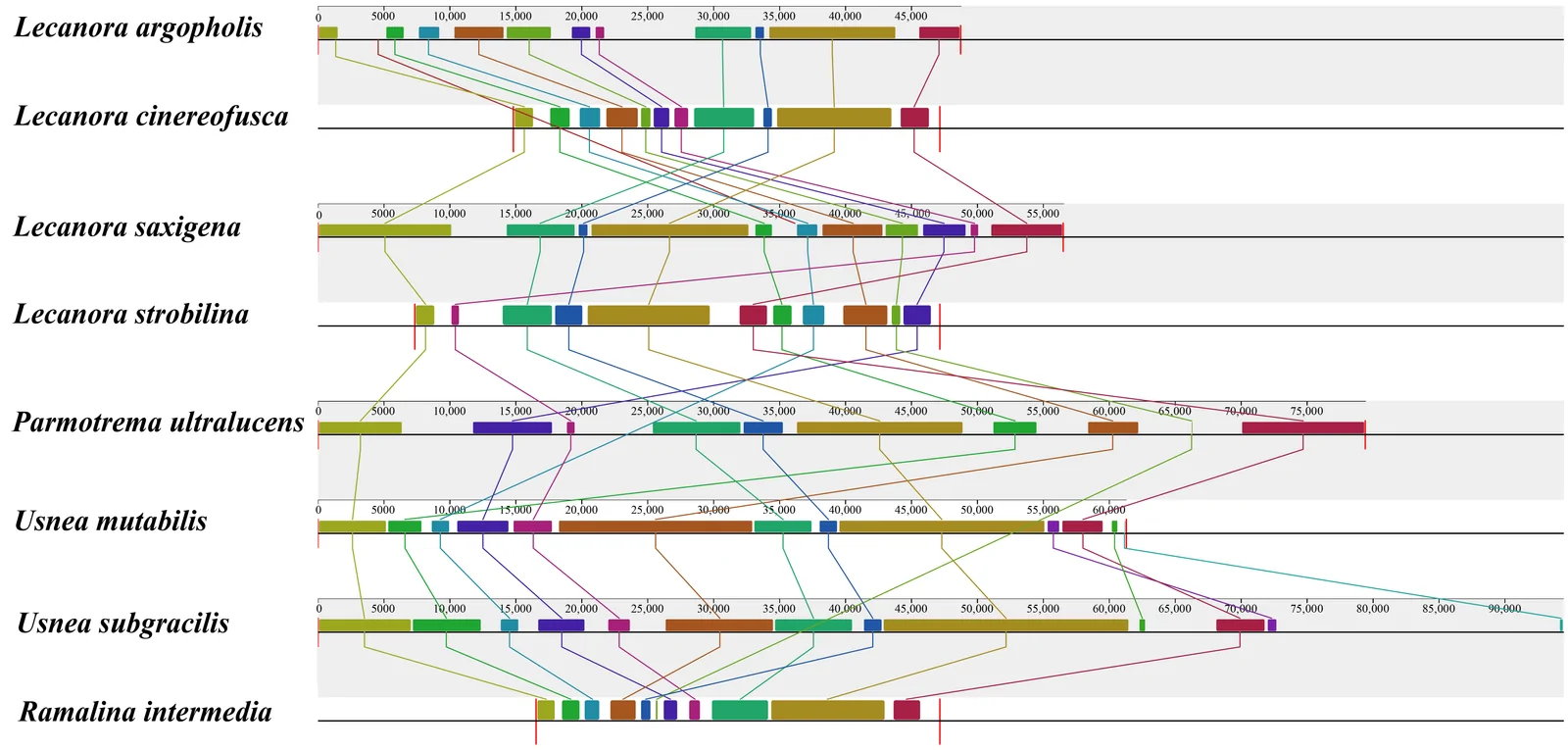
Comparative synteny of eight lichen-forming fungal mitochondrial genomes. Circular genomes were normalized to a common *cox1* starting position while preserving their original orientation. Colored blocks represent homologous locally collinear blocks (LCBs), and connecting lines indicate correspondence of homologous regions among the compared mitogenomes. Red vertical lines indicate the start/end boundaries of the linearized genome displays after normalization to a common *cox1* starting position.

**Figure 6:**
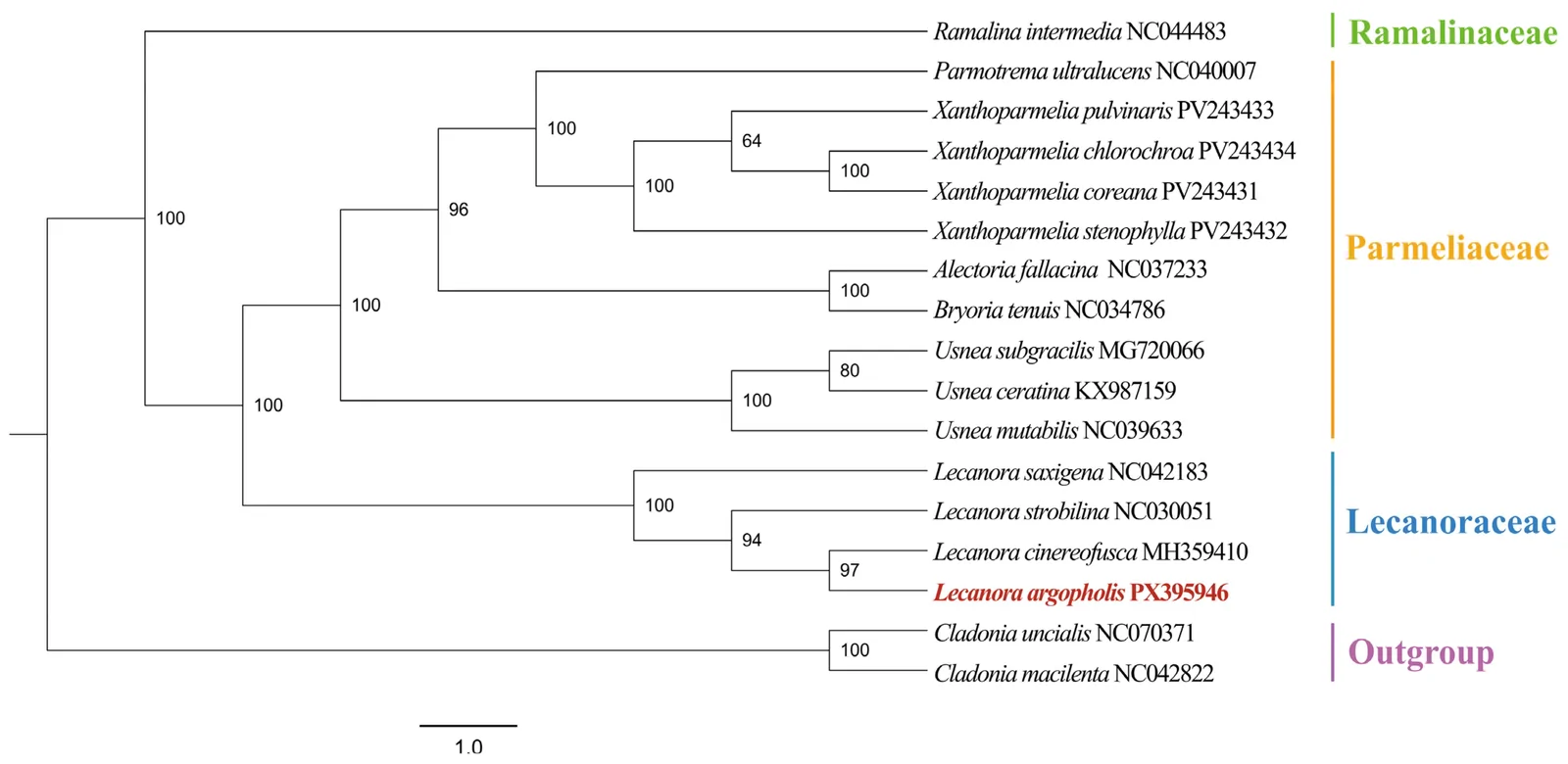
Maximum-likelihood phylogeny of 17 lichen-forming fungal taxa inferred from 14 concatenated mitochondrial protein-coding genes. Numbers at the nodes indicate ultrafast bootstrap support values based on 1000 replicates. *Lecanora argopholis* is highlighted in red. *Cladonia uncialis* and *C. macilenta* were designated as outgroups, and the tree was rooted accordingly. The scale bar represents substitutions per site. Colored vertical bars and labels indicate the corresponding taxonomic groups: Ramalinaceae (green), Parmeliaceae (orange), Lecanoraceae (blue), and the outgroup (purple).

**Table 1:** Functional classification of genes in the mitochondrial genome of *Lecanora argopholis*.

Functional Category	Genes	No. of Genes
Complex I (NADH dehydrogenase)	*nad1*, *nad2*, *nad3*, *nad4*, *nad4L*, *nad5*, *nad6*	7
Complex III (ubiquinol–cytochrome c reductase)	*cob*	1
Complex IV (cytochrome c oxidase)	*cox1*, *cox2*, *cox3*	3
ATP synthase	*atp6*, *atp8*	2
Ribosomal protein	*rps3*	1
Ribosomal RNAs	*rrnS*, *rrnL*	2
Transfer RNAs	*trnA*, *trnC*, *trnD*, *trnE*, *trnF*, *trnG*, *trnH*, *trnI*, *trnK* (×2), *trnL1*, *trnL2*, *trnM* (×3), *trnN*, *trnP*, *trnQ*, *trnR* (×3), *trnS1*, *trnS2*, *trnT*, *trnV* (×3), *trnW*, *trnY*	29
Total		45

## Data Availability

The genome sequence data that support the findings of this study are openly available in GenBank of NCBI under the accession number PX395946. The associated BioProject, SRA and BioSample numbers are PRJNA1294799, SRR34657009, and SAMN50125876, respectively.

## Supplementary Materials

The following supporting information can be downloaded at: https://www.mdpi.com/article/10.3390/jof12100714/s1, Figure S1: Sequencing depth across the mitochondrial genome of *Lecanora argopholis*; Table S1: Specimen collection information for *Lecanora argopholis*; Table S2: Species information, GenBank accession numbers, and inclusion in the phylogenetic and comparative mitochondrial analyses; Table S3: Comparative mitochondrial tRNA analysis among four *Lecanora* species; Table S4: Comparative relative synonymous codon usage (RSCU) among four *Lecanora* species; Table S5: Comparative mitochondrial synteny and gene-order analysis among eight lichen-forming fungi.
