# Supplementary material for: The First Complete Mitochondrial Genome of Lecanora argopholis and Its Phylogenetic Implications for Lichen-Forming Fungi
Source: J Fungi (Basel). 2026 Sep 23;12(10):714. doi: 10.3390/jof12100714 (PMC13705890; doi:10.3390/jof12100714)
Supplement: Supplementary file 1 [file jof-12-00714-s001.zip › Supplementary Figure.pdf]

Supplementary Figure S1

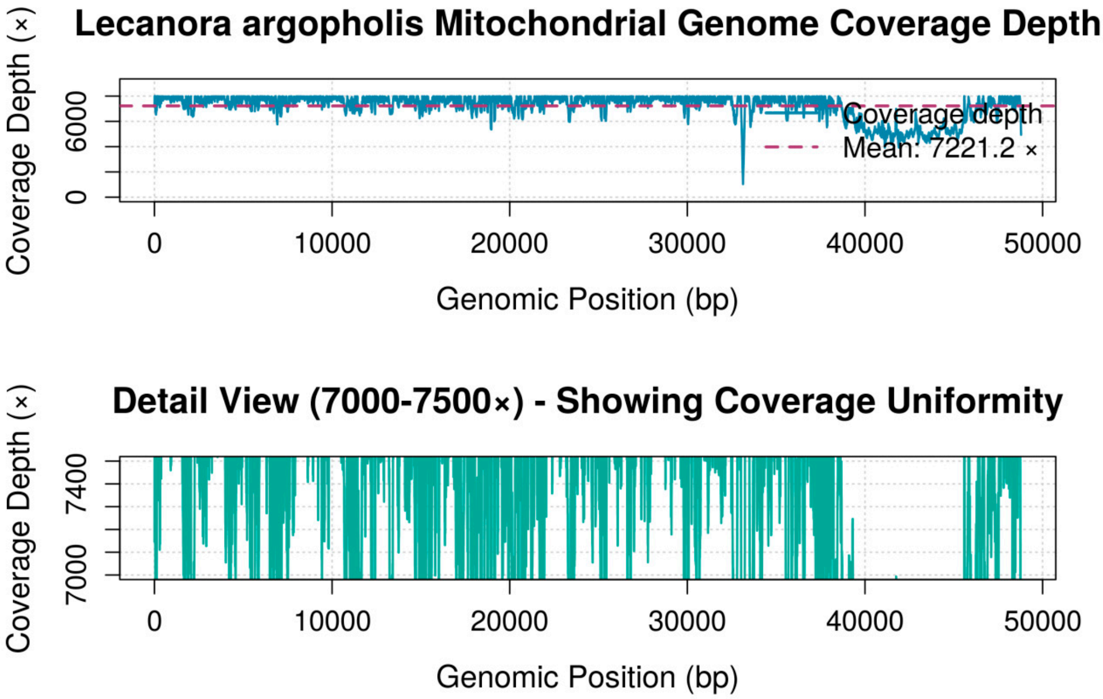

Figure S1. Sequencing depth across the mitochondrial genome of *Lecanora argopholis*. The complete mitogenome (48,790 bp) showed generally high coverage, with an average sequencing depth of 7,221× (range: 1,032–8,015×) and 100% coverage. Sequencing depth was generally consistent across most genomic regions, although local fluctuations were observed. The red dashed line indicates the mean sequencing depth.
