# Supplementary material for: The First Complete Mitochondrial Genome of Lecanora argopholis and Its Phylogenetic Implications for Lichen-Forming Fungi
Source: J Fungi (Basel). 2026 Sep 23;12(10):714. doi: 10.3390/jof12100714 (PMC13705890; doi:10.3390/jof12100714)
Supplement: Supplementary file 1 [file jof-12-00714-s001.zip › Supplementary Tables.pdf]

Supplementary Materials

Supplementary Tables

Supplementary Table S1. Specimen collection information for *Lecanora argopholis*.

Species	Latitude	Longitude	Elevation
Lecanora argopholis	43°01′11″ N	83°24′47″ E	2865 m

Supplementary Table S2. Species information, GenBank accession numbers, and inclusion in the phylogenetic and comparative mitochondrial analyses.

Family	Species	GenBank accession	Phylogeny	Synteny	tRNA	RSCU
Ramalinaceae	*Ramalina intermedia*	NC_044483	Yes	Yes	—	—
Parmeliaceae	*Parmotrema ultralucens*	NC_040007	Yes	Yes	—	—
Parmeliaceae	*Xanthoparmelia pulvinaris*	PV243433	Yes	—	—	—
Parmeliaceae	*Xanthoparmelia chlorochroa*	PV243434	Yes	—	—	—
Parmeliaceae	*Xanthoparmelia coreana*	PV243431	Yes	—	—	—
Parmeliaceae	*Xanthoparmelia stenophylla*	PV243432	Yes	—	—	—
Parmeliaceae	*Alectoria fallacina*	NC_037233	Yes	—	—	—
Parmeliaceae	*Bryoria tenuis*	NC_034786	Yes	—	—	—
Parmeliaceae	*Usnea subgracilis*	MG720066	Yes	Yes	—	—
Parmeliaceae	*Usnea ceratina*	KX987159	Yes	—	—	—
Parmeliaceae	*Usnea mutabilis*	NC_039633	Yes	Yes	—	—
Lecanoraceae	*Lecanora saxigena*	NC_042183	Yes	Yes	Yes	Yes
Lecanoraceae	*Lecanora strobilina*	NC_030051	Yes	Yes	Yes	Yes
Lecanoraceae	*Lecanora cinereofusca*	MH359410	Yes	Yes	Yes	Yes
Lecanoraceae	*Lecanora argopholis*	PX395946	Yes	Yes	Yes	Yes
Cladoniaceae	*Cladonia uncialis*	NC_070371	Yes	—	—	—
Cladoniaceae	*Cladonia macilenta*	NC_042822	Yes	—	—	—

Supplementary Table S3. Comparative mitochondrial tRNA analysis among four *Lecanora* species.

Table S3A. Comparative mitochondrial tRNAs identified in four *Lecanora* species.

Species	Accession	tRNA	Anticodon (5′–3′)	Copy	Strand	Length (nt)	G–U pairs	Annotation/QC note
Lecanora argopholis	PX395946	*trnA*	UGC	1/1	+	72	2	
Lecanora cinereofusca	MH359410	*trnA*	UGC	1/1	+	72	2	
Lecanora saxigena	NC_042183	*trnA*	UGC	1/1	+	72	2	
Lecanora strobilina	NC_030051	*trnA*	UGC	1/1	+	72	2	
Lecanora argopholis	PX395946	*trnC*	GCA	1/1	+	70	2	
Lecanora cinereofusca	MH359410	*trnC*	GCA	1/1	+	70	1	
Lecanora saxigena	NC_042183	*trnC*	GCA	1/1	+	71	0	
Lecanora strobilina	NC_030051	*trnC*	GCA	1/1	+	70	2	
Lecanora argopholis	PX395946	*trnD*	GUC	1/1	+	73	1	
Lecanora cinereofusca	MH359410	*trnD*	GUC	1/1	+	73	0	
Lecanora saxigena	NC_042183	*trnD*	GUC	1/1	+	73	2	
Lecanora strobilina	NC_030051	*trnD*	GUC	1/1	+	73	1	
Lecanora argopholis	PX395946	*trnE*	UUC	1/1	+	71	2	
Lecanora cinereofusca	MH359410	*trnE*	UUC	1/1	+	71	2	
Lecanora saxigena	NC_042183	*trnE*	UUC	1/1	+	71	1	
Lecanora strobilina	NC_030051	*trnE*	UUC	1/1	+	71	1	
Lecanora argopholis	PX395946	*trnF*	GAA	1/1	+	73	3	
Lecanora cinereofusca	MH359410	*trnF*	GAA	1/1	+	73	2	
Lecanora saxigena	NC_042183	*trnF*	GAA	1/1	+	73	2	
Lecanora strobilina	NC_030051	*trnF*	GAA	1/1	+	73	2	
Lecanora argopholis	PX395946	*trnG*	UCC	1/1	+	71	2	
Lecanora cinereofusca	MH359410	*trnG*	UCC	1/1	+	71	1	
Lecanora saxigena	NC_042183	*trnG*	UCC	1/1	+	71	1	
Lecanora strobilina	NC_030051	*trnG*	UCC	1/1	+	71	1	

Species	Accession	tRNA	Anticodon (5′–3′)	Copy	Strand	Length (nt)	G–U pairs	Annotation/QC note
Lecanora argopholis	PX395946	*trnH*	GUG	1/1	+	73	1	
Lecanora cinereofusca	MH359410	*trnH*	GUG	1/1	+	73	1	
Lecanora saxigena	NC_042183	*trnH*	GUG	1/1	+	72	2	
Lecanora strobilina	NC_030051	*trnH*	GUG	1/1	+	73	1	
Lecanora argopholis	PX395946	*trnI*	GAU	1/1	+	72	3	
Lecanora cinereofusca	MH359410	*trnI*	GAU	1/1	+	72	3	
Lecanora saxigena	NC_042183	*trnI*	GAU	1/1	+	72	4	
Lecanora strobilina	NC_030051	*trnI*	GAU	1/1	+	72	4	
Lecanora argopholis	PX395946	*trnK*	UUU	1/2	+	73	5	
Lecanora argopholis	PX395946	*trnK*	UUU	2/2	+	71	0	
Lecanora cinereofusca	MH359410	*trnK*	UUU	1/1	+	73	5	
Lecanora saxigena	NC_042183	*trnK*	UUU	1/2	+	73	4	
Lecanora saxigena	NC_042183	*trnK*	UUU	2/2	+	73	4	
Lecanora strobilina	NC_030051	*trnK*	UUU	1/1	+	73	5	
Lecanora argopholis	PX395946	*trnL1*	UAG	1/1	+	84	1	
Lecanora cinereofusca	MH359410	*trnL1*	UAG	1/1	+	82	2	
Lecanora saxigena	NC_042183	*trnL1*	UAG	1/1	+	82	0	
Lecanora strobilina	NC_030051	*trnL1*	UAG	1/1	+	85	2	
Lecanora argopholis	PX395946	*trnL2*	UAA	1/1	+	83	2	
Lecanora cinereofusca	MH359410	*trnL2*	UAA	1/1	+	83	3	
Lecanora saxigena	NC_042183	*trnL2*	UAA	1/1	+	83	3	
Lecanora strobilina	NC_030051	*trnL2*	UAA	1/1	+	83	2	
Lecanora argopholis	PX395946	*trnM*	CAU	1/3	+	71	2	
Lecanora argopholis	PX395946	*trnM*	CAU	2/3	+	73	3	
Lecanora argopholis	PX395946	*trnM*	CAU	3/3	+	73	0	
Lecanora cinereofusca	MH359410	*trnM*	CAU	1/3	+	71	2	

Species	Accession	tRNA	Anticodon (5′–3′)	Copy	Strand	Length (nt)	G–U pairs	Annotation/QC note
Lecanora cinereofusca	MH359410	*trnM*	CAU	2/3	+	73	4	
Lecanora cinereofusca	MH359410	*trnM*	CAU	3/3	+	73	1	
Lecanora saxigena	NC_042183	*trnM*	CAU	1/3	+	72	1	
Lecanora saxigena	NC_042183	*trnM*	CAU	2/3	+	73	4	
Lecanora saxigena	NC_042183	*trnM*	CAU	3/3	+	73	1	
Lecanora strobilina	NC_030051	*trnM*	CAU	1/3	+	71	2	
Lecanora strobilina	NC_030051	*trnM*	CAU	2/3	+	73	5	
Lecanora strobilina	NC_030051	*trnM*	CAU	3/3	+	73	1	
Lecanora argopholis	PX395946	*trnN*	GUU	1/1	+	71	1	Spans the circular-genome origin; validated after genome rotation.
Lecanora cinereofusca	MH359410	*trnN*	GUU	1/1	+	71	2	
Lecanora saxigena	NC_042183	*trnN*	GUU	1/1	+	71	1	
Lecanora argopholis	PX395946	*trnP*	UGG	1/1	+	73	1	
Lecanora cinereofusca	MH359410	*trnP*	UGG	1/1	+	73	2	
Lecanora saxigena	NC_042183	*trnP*	UGG	1/1	+	73	1	
Lecanora strobilina	NC_030051	*trnP*	UGG	1/1	+	73	1	
Lecanora argopholis	PX395946	*trnQ*	UUG	1/1	+	73	0	
Lecanora cinereofusca	MH359410	*trnQ*	UUG	1/1	+	73	0	
Lecanora saxigena	NC_042183	*trnQ*	UUG	1/1	+	73	0	
Lecanora strobilina	NC_030051	*trnQ*	UUG	1/1	+	73	1	
Lecanora cinereofusca	MH359410	*trnR*	ACG	1/1	+	73	0	
Lecanora argopholis	PX395946	*trnR*	CCU	1/1	+	71	1	
Lecanora cinereofusca	MH359410	*trnR*	CCU	1/1	+	71	2	
Lecanora saxigena	NC_042183	*trnR*	CCU	1/1	+	71	2	
Lecanora strobilina	NC_030051	*trnR*	CCU	1/1	+	71	1	
Lecanora argopholis	PX395946	*trnR*	UCG	1/1	+	73	0	
Lecanora saxigena	NC_042183	*trnR*	UCG	1/1	+	73	1	

Species	Accession	tRNA	Anticodon (5′–3′)	Copy	Strand	Length (nt)	G–U pairs	Annotation/QC note
Lecanora strobilina	NC_030051	*trnR*	UCG	1/1	+	73	0	
Lecanora argopholis	PX395946	*trnR*	UCU	1/1	+	71	3	
Lecanora cinereofusca	MH359410	*trnR*	UCU	1/1	+	71	2	
Lecanora saxigena	NC_042183	*trnR*	UCU	1/1	+	71	1	
Lecanora strobilina	NC_030051	*trnR*	UCU	1/1	+	71	1	
Lecanora argopholis	PX395946	*trnS1*	GCU	1/1	+	82	3	
Lecanora cinereofusca	MH359410	*trnS1*	GCU	1/1	+	82	1	
Lecanora saxigena	NC_042183	*trnS1*	GCU	1/1	+	82	3	
Lecanora strobilina	NC_030051	*trnS1*	GCU	1/1	+	82	2	
Lecanora argopholis	PX395946	*trnS2*	UGA	1/1	+	86	3	
Lecanora cinereofusca	MH359410	*trnS2*	UGA	1/1	+	86	2	
Lecanora saxigena	NC_042183	*trnS2*	UGA	1/1	+	86	3	
Lecanora strobilina	NC_030051	*trnS2*	UGA	1/1	+	86	3	GenBank annotation conflict; direct sequence inspection confirmed the UGA anticodon assignment.
Lecanora argopholis	PX395946	*trnT*	UGU	1/1	+	71	3	
Lecanora cinereofusca	MH359410	*trnT*	UGU	1/1	+	71	3	
Lecanora saxigena	NC_042183	*trnT*	UGU	1/1	+	71	3	
Lecanora strobilina	NC_030051	*trnT*	UGU	1/1	+	71	3	
Lecanora argopholis	PX395946	*trnV*	UAC	1/3	+	72	0	
Lecanora argopholis	PX395946	*trnV*	UAC	2/3	+	72	0	
Lecanora argopholis	PX395946	*trnV*	UAC	3/3	+	72	0	
Lecanora cinereofusca	MH359410	*trnV*	UAC	1/1	+	72	1	
Lecanora saxigena	NC_042183	*trnV*	UAC	1/1	+	72	1	
Lecanora strobilina	NC_030051	*trnV*	UAC	1/1	+	72	1	
Lecanora argopholis	PX395946	*trnW*	UCA	1/1	+	71	0	
Lecanora cinereofusca	MH359410	*trnW*	UCA	1/1	+	71	0	
Lecanora saxigena	NC_042183	*trnW*	UCA	1/1	+	71	1	

Species	Accession	tRNA	Anticodon (5′–3′)	Copy	Strand	Length (nt)	G–U pairs	Annotation/QC note
Lecanora strobilina	NC_030051	*trnW*	UCA	1/1	+	71	1	
Lecanora argopholis	PX395946	*trnY*	GUA	1/1	+	85	1	
Lecanora cinereofusca	MH359410	*trnY*	GUA	1/1	+	85	0	
Lecanora saxigena	NC_042183	*trnY*	GUA	1/1	+	85	0	
Lecanora strobilina	NC_030051	*trnY*	GUA	1/1	+	85	3	

Notes: 1. All four mitochondrial genomes were analyzed using tRNAscan-SE v2.0.12 with the same organellar search setting. Only confidently retained predictions are included; three low-confidence predictions were excluded from comparative structural inference.

2. Anticodons are reported in RNA notation (5′–3′), and copy number is reported as copy index/total confident copy number. G–U pairs were counted from predicted tRNAscan-SE secondary structures; no other noncanonical paired bases were detected among the 107 confidently retained tRNAs.

3. Raw Sup/TCA predictions were standardized as *trnW(UCA)* for comparison under fungal mitochondrial genetic code 4 and in accordance with the corresponding GenBank annotation. Additional locus-specific QC information is provided in the Annotation/QC note column.

Table S3B. Comparative structural features of five focal mitochondrial tRNAs in four *Lecanora* species.

Focal tRNA	Anticodon (5′–3′)	*L. argopholis* Length / paired bp / G–U	*L. cinereofusca* Length / paired bp / G–U	*L. saxigena* Length / paired bp / G–U	*L. strobilina* Length / paired bp / G–U	Structural interpretation	Local genomic context
trnS1	GCU	82 / 23 / 3	82 / 25 / 1	82 / 23 / 3	82 / 25 / 2	Conserved length with shared local pairing variation.	Immediate and wider five-feature neighborhoods conserved in all four taxa.
trnS2	UGA	86 / 25 / 3	86 / 25 / 2	86 / 24 / 3	86 / 24 / 3	Conserved length with shared local pairing variation.	Immediate and wider five-feature neighborhoods conserved in all four taxa.
trnL1	UAG	84 / 24 / 1	82 / 25 / 2	82 / 24 / 0	85 / 23 / 2	Variable length and local pairing configuration among the four sampled taxa.	Immediate and wider five-feature neighborhoods conserved in all four taxa.
trnL2	UAA	83 / 23 / 2	83 / 23 / 3	83 / 22 / 3	83 / 23 / 2	Conserved length with minor local pairing variation.	Immediate and wider five-feature neighborhoods conserved in all four taxa.
trnY	GUA	85 / 24 / 1	85 / 24 / 0	85 / 23 / 0	85 / 26 / 3	Conserved length with distinct local pairing patterns among the four sampled taxa.	Immediate neighborhood conserved in all four taxa; wider five-feature context variable among taxa.

Notes: 1. *trnS1*, *trnS2*, *trnL1*, *trnL2*, and *trnY* were selected for focused structural comparison among the four sampled *Lecanora* species.
2. No clear correspondence was observed between focal tRNA structural variation and local mitochondrial gene-order differences.

Supplementary Table S4. Comparative relative synonymous codon usage (RSCU) among four *Lecanora* species.

Table S4A. Species-level summary of comparative mitochondrial codon usage among four *Lecanora* species.

Species	Accession	PCGs used (n)	Total sense codons	Codons with RSCU > 1.0	A-ending preferential codons	U-ending preferential codons	G-ending preferential codons	C-ending preferential codons	A/U-ending preferential fraction	Observed A/U-ending codon proportion	UUA count	UUA RSCU
Lecanora argopholis	PX395946	13	5252	25	12	13	0	0	1.000	0.797	418	3.344
Lecanora cinereofusca	MH359410	13	4598	26	12	13	1	0	0.962	0.769	389	3.353
Lecanora saxigena	NC_042183	13	4597	26	12	14	0	0	1.000	0.813	413	3.540
Lecanora strobilina	NC_030051	13	4979	24	11	13	0	0	1.000	0.803	432	3.498

Table S4B. Relative synonymous codon usage (RSCU) values for 62 sense codons in four *Lecanora* mitochondrial genomes.

Amino acid	Codon	Third base	*L. argopholis* RSCU	*L. cinereofusca* RSCU	*L. saxigena* RSCU	*L. strobilina* RSCU
Phe	UUU	U	1.682	1.597	1.808	1.752
Phe	UUC	C	0.318	0.403	0.192	0.248
Leu	UUA	A	3.344	3.353	3.540	3.498
Leu	UUG	G	0.672	0.629	0.591	0.696
Leu	CUU	U	0.824	0.716	0.874	0.858
Leu	CUC	C	0.088	0.121	0.086	0.097
Leu	CUA	A	0.864	0.922	0.814	0.632
Leu	CUG	G	0.208	0.259	0.094	0.219
Ile	AUU	U	0.931	0.862	1.004	0.938
Ile	AUC	C	0.322	0.351	0.352	0.290
Ile	AUA	A	1.747	1.787	1.644	1.772
Met	AUG	G	1.000	1.000	1.000	1.000
Val	GUU	U	1.844	1.723	1.718	1.833
Val	GUC	C	0.274	0.201	0.220	0.260
Val	GUA	A	1.346	1.535	1.471	1.325
Val	GUG	G	0.536	0.541	0.591	0.582
Ser	UCU	U	1.864	1.656	2.009	2.014
Ser	UCC	C	0.321	0.524	0.443	0.359
Ser	UCA	A	1.210	1.358	1.095	1.228
Ser	UCG	G	0.173	0.184	0.180	0.138
Ser	AGU	U	1.827	1.627	1.774	1.628
Ser	AGC	C	0.605	0.651	0.499	0.634
Pro	CCU	U	2.423	2.122	2.113	2.497
Pro	CCC	C	0.320	0.585	0.528	0.508
Pro	CCA	A	1.006	1.049	1.208	0.906
Pro	CCG	G	0.251	0.244	0.151	0.088
Thr	ACU	U	1.779	1.696	1.631	1.606

Amino acid	Codon	Third base	*L. argopholis* RSCU	*L. cinereofusca* RSCU	*L. saxigena* RSCU	*L. strobilina* RSCU
Thr	ACC	C	0.384	0.342	0.412	0.296
Thr	ACA	A	1.737	1.681	1.751	1.873
Thr	ACG	G	0.100	0.280	0.206	0.225
Ala	GCU	U	2.196	1.952	1.895	1.931
Ala	GCC	C	0.503	0.751	0.519	0.514
Ala	GCA	A	1.175	1.078	1.291	1.389
Ala	GCG	G	0.126	0.218	0.295	0.167
Tyr	UAU	U	1.418	1.285	1.557	1.487
Tyr	UAC	C	0.582	0.715	0.443	0.513
His	CAU	U	1.402	1.195	1.461	1.510
His	CAC	C	0.598	0.805	0.539	0.490
Gln	CAA	A	1.481	1.633	1.609	1.621
Gln	CAG	G	0.519	0.367	0.391	0.379
Asn	AAU	U	1.338	1.160	1.438	1.289
Asn	AAC	C	0.662	0.840	0.562	0.711
Lys	AAA	A	1.672	1.633	1.562	1.686
Lys	AAG	G	0.328	0.367	0.438	0.314
Asp	GAU	U	1.514	1.383	1.600	1.570
Asp	GAC	C	0.486	0.617	0.400	0.430
Glu	GAA	A	1.413	1.417	1.612	1.563
Glu	GAG	G	0.587	0.583	0.388	0.437
Cys	UGU	U	1.677	1.625	1.833	1.667
Cys	UGC	C	0.323	0.375	0.167	0.333
Trp	UGA	A	1.662	1.803	1.770	1.836
Trp	UGG	G	0.338	0.197	0.230	0.164
Arg	CGU	U	0.980	0.550	0.872	0.773
Arg	CGC	C	0.235	0.055	0.051	0.136
Arg	CGA	A	0.275	0.330	0.359	0.273
Arg	CGG	G	0.078	0.000	0.103	0.045

**Amino acid**	**Codon**	**Third base**	***L. argopholis*** **RSCU**	***L. cinereofusca*** **RSCU**	***L. saxigena*** **RSCU**	***L. strobilina*** **RSCU**
Arg	AGA	A	3.647	4.018	3.949	4.000
Arg	AGG	G	0.784	1.046	0.667	0.773
Gly	GGU	U	2.403	2.303	2.481	2.479
Gly	GGC	C	0.218	0.339	0.301	0.225
Gly	GGA	A	0.969	0.989	0.947	0.958
Gly	GGG	G	0.410	0.369	0.271	0.338

Notes: 1. Comparative RSCU values were calculated from 13 mitochondrial PCGs shared by all four *Lecanora* taxa (*atp6*, *atp8*, *cob*, *cox1*, *cox2*, *cox3*, *nad1*, *nad2*, *nad3*, *nad4*, *nad5*, *nad6*, and *rps3*) under NCBI mitochondrial translation table 4.

2. Under translation table 4, UGA encodes tryptophan; UAA and UAG were excluded as stop codons, yielding 62 sense codons. Preferential codons were defined as RSCU > 1.0.

3. The A/U-ending preferential fraction is the proportion of A- or U-ending codons among preferential codons, whereas the observed A/U-ending codon proportion is their proportion among all observed sense codons.

Supplementary Table S5. Comparative mitochondrial synteny and gene-order analysis among eight lichen-forming fungi.

Table S5A. Pairwise mitochondrial gene-order similarity among eight lichen-forming fungi.

Taxon A	Accession A	Taxon B	Accession B	Conserved adjacencies	Conserved adjacency fraction	Adjacency Jaccard similarity
Lecanora argopholis	PX395946	*Lecanora cinereofusca*	MH359410	15/15	1.000	1.000
Lecanora argopholis	PX395946	*Lecanora saxigena*	NC_042183	12/15	0.800	0.667
Lecanora argopholis	PX395946	*Lecanora strobilina*	NC_030051	12/15	0.800	0.667
Lecanora argopholis	PX395946	*Parmotrema ultralucens*	NC_040007	9/15	0.600	0.429
Lecanora argopholis	PX395946	*Usnea mutabilis*	NC_039633	12/15	0.800	0.667
Lecanora argopholis	PX395946	*Usnea subgracilis*	MG720066	12/15	0.800	0.667
Lecanora argopholis	PX395946	*Ramalina intermedia*	NC_044483	12/15	0.800	0.667
Lecanora cinereofusca	MH359410	*Lecanora saxigena*	NC_042183	12/15	0.800	0.667
Lecanora cinereofusca	MH359410	*Lecanora strobilina*	NC_030051	12/15	0.800	0.667
Lecanora cinereofusca	MH359410	*Parmotrema ultralucens*	NC_040007	9/15	0.600	0.429
Lecanora cinereofusca	MH359410	*Usnea mutabilis*	NC_039633	12/15	0.800	0.667
Lecanora cinereofusca	MH359410	*Usnea subgracilis*	MG720066	12/15	0.800	0.667
Lecanora cinereofusca	MH359410	*Ramalina intermedia*	NC_044483	12/15	0.800	0.667
Lecanora saxigena	NC_042183	*Lecanora strobilina*	NC_030051	10/15	0.667	0.500
Lecanora saxigena	NC_042183	*Parmotrema ultralucens*	NC_040007	10/15	0.667	0.500
Lecanora saxigena	NC_042183	*Usnea mutabilis*	NC_039633	9/15	0.600	0.429
Lecanora saxigena	NC_042183	*Usnea subgracilis*	MG720066	9/15	0.600	0.429
Lecanora saxigena	NC_042183	*Ramalina intermedia*	NC_044483	9/15	0.600	0.429
Lecanora strobilina	NC_030051	*Parmotrema ultralucens*	NC_040007	7/15	0.467	0.304
Lecanora strobilina	NC_030051	*Usnea mutabilis*	NC_039633	9/15	0.600	0.429
Lecanora strobilina	NC_030051	*Usnea subgracilis*	MG720066	9/15	0.600	0.429
Lecanora strobilina	NC_030051	*Ramalina intermedia*	NC_044483	9/15	0.600	0.429
Parmotrema ultralucens	NC_040007	*Usnea mutabilis*	NC_039633	8/15	0.533	0.364
Parmotrema ultralucens	NC_040007	*Usnea subgracilis*	MG720066	8/15	0.533	0.364
Parmotrema ultralucens	NC_040007	*Ramalina intermedia*	NC_044483	7/15	0.467	0.304

Taxon A	Accession A	Taxon B	Accession B	Conserved adjacencies	Conserved adjacency fraction	Adjacency Jaccard similarity
Usnea mutabilis	NC_039633	*Usnea subgracilis*	MG720066	15/15	1.000	1.000
Usnea mutabilis	NC_039633	*Ramalina intermedia*	NC_044483	10/15	0.667	0.500
Usnea subgracilis	MG720066	*Ramalina intermedia*	NC_044483	10/15	0.667	0.500

Note: Gene-order comparisons were based on 15 mitochondrial genes shared by all eight taxa (13 protein-coding genes plus *rrnS* and *rrnL*). Conserved adjacency fraction = shared circular adjacencies / 15. Adjacency Jaccard similarity = $|A \cap B| / |A \cup B|$.

Table S5B. Universal and major partially shared locally collinear blocks (LCBs) identified by progressiveMauve.

LCB ID	XMFA block	Presence	Representative mitochondrial gene region in *Lecanora argopholis*	Orientation	Taxa absent	Note
CoreLCB01	2	8/8	*cox1*	+	None	—
CoreLCB02	3	8/8	*nad4*	+	None	—
CoreLCB03	7	8/8	*cob–cox2*	+	None	—
CoreLCB04	5	8/8	*nad1*	+	None	—
CoreLCB05	6	8/8	*atp6*	+	None	—
CoreLCB06	9	8/8	*nad6–atp8–rrnS* region	+	None	—
CoreLCB07	10	8/8	*cox3*	+	None	—
CoreLCB08	13	8/8	*rrnL–nad2–nad3* region	+	None	—
CoreLCB09	12	8/8	*nad4L–nad5* region	+	None	—
PartialLCB01	4	7/8	*rps3*	+	*Parmotrema ultralucens*	The *rps3*-associated shared LCB was not recovered in *P. ultralucens*.

Note: Representative gene-region labels summarize the principal annotated mitochondrial features overlapping each LCB in *L. argopholis*; LCB boundaries do not necessarily coincide with individual gene boundaries. "+" indicates forward orientation, and no reverse-oriented shared LCBs were detected. The table summarizes the nine universal core LCBs and the major *rps3*-associated partial LCB; the complete comparison identified 15 shared LCBs in total, including nine universal and six partial LCBs. Absence of the *rps3*-associated shared LCB in *P. ultralucens* does not indicate absence of the annotated *rps3* gene.
